# Automatic software for extracellular volume fraction mapping in the myocardium

**DOI:** 10.1186/1532-429X-17-S1-W34

**Published:** 2015-02-03

**Authors:** Luisa Altabella, Cristian Borrazzo, Marco Carnì, Nicola Galea, Elisabetta Di Castro, Marco Francone, Carlo Catalano, Iacopo Carbone

**Affiliations:** 1 Department of Molecular Medicine, Sapienza University of Rome, Rome, Italy; 2 Medical Physics unit, Policlinico Umberto I, Rome, Italy; 3 Department of Radiological Sciences, Oncology and Pathology, Sapienza University of Rome, Rome, Italy

## Background

CMR is a useful tool for myocardial tissue characterization and to assess fibrosis and edema non-invasively. The estimation of extracellular volume fraction (ECV) is emerging as accurate biomarkers in many cardiac diseases associated with diffuse myocardial fibrosis. In this work, we present our automatic tool for ECV map creation. All the computational system consists of an executable file developed in Matlab (Mathworks Inc.).

## Methods

30 subjects underwent CMR on a 1.5T MR scanner (Avanto, Siemens). T1 quantification was performed with a Modified Look-Locker Inversion-recovery (MOLLI) sequence before and 15 minutes after a 0.1mmol/kg intravenous bolus of gadobenate dimeglumine (Gd-BOPTA; Multihance^©^, Bracco). Imaging parameters were: matrix 218×256, voxel size 1.41x1.41x8 mm^3^, TR/TE 1.44/1.12 ms, minimum inversion time 120 ms, time increment 80 ms, flip angle 35°. The protocol used is 5(3)3. Myocardial ECV maps were generated as follow. All MOLLI images were motion corrected within each series. These images were co-registrated using an affine image registration between pre-contrast image and post-contrast image with longest inversion time to avoid possibly patient position variations. Then the image transformation was applied to the whole post contrast series. Motion corrected and co-registered images were used to generate pre and post contrast T1 mapping. T1 time was calculated with a 3-parameter curve fitting using a Levenberg-Marquardt algorithm and T1* correction. Pixel-wise ECV map was computed following the relation: ECV=[1-hematocrit]*ΔR1_myo_/ΔR1_blood_ where ΔR1_myo_=(1/T1_myo-post_)-(1/T1_myo-pre_) was obtained taking the reciprocal of the T1 maps on a pixel-by-pixel basis. The blood relaxation rate ΔR1_blood_=(1/T1_blood-post_)-(1/T1_blood-pre_) was calculated automatically creating a mask on the T1 pre contrast map applying a threshold on pixels with T1 greater than 1250 ms (corrected for partial volume effects). The mask was then applied on T1 post-contrast map to calculate the mean T1_blood-post_.

## Results

Comparison between blood T1 values obtained automatically using the mask and those obtained with manual segmentation shown a significant correlation both for pre (r=0.84 p<0.01) and post (r=0.99 p<0.01) contrast datasets. Then we tested the myocardium ECV values. Also for myocardium ECV values calculated by two approaches, a significant correlation (r=0.95 p<0.01) was found.

## Conclusions

This work demonstrated that it is possible to obtain informative ECV maps using our software. Comparing with ECV value from manually drawn ROI in myocardium in the T1 and T2 maps, the ECV maps are in agreement with the standard approach to ECV calculation. Furthermore, pixel-wise ECV maps, obtained with this automatic software, allow to directly visualize the extent and severity of ECV alterations respect to manual approach.

**Figure 1:**
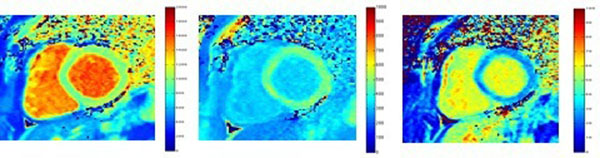
T1 pre contrast map (ms), T1 post contrast map (ms) and ECV map (%) obtained with our software

